# Performing lifestyle changes and reducing stroke risk: a process evaluation of a stroke prevention intervention

**DOI:** 10.1136/bmjopen-2026-123012

**Published:** 2026-09-30

**Authors:** Cecilia Johnsson, Eric Asaba, Susanne Guidetti, Maria Hagströmer, Ann-Helen Patomella

**Affiliations:** 1 Department of Neurobiology, Care Sciences and Society (NVS), Division of Occupational Therapy, Karolinska Institute, Stockholm, Sweden; 2 Department of Health and Rehabilitation, Sahlgrenska Academy, Gothenburg, Sweden; 3 Medical Unit Allied Health Professionals, Womens Health Professionals Theme, Karolinska University Hospital, Stockholm, Sweden; 4 Department of Neurobiology Care Sciences and Society (NVS), Division of Physiotherapy, Karolinska Institute, Stockholm, Sweden; 5 Academic Primary Care Health Centre, Region Stockholm, Stockholm, Sweden

**Keywords:** Cardiovascular Disease, eHealth, Health Literacy, Patient-Centered Care, Primary Health Care, Primary Prevention

## Abstract

**Objective:**

To examine the implementation process, mechanisms of change and contextual influences of a stroke prevention intervention.

**Design:**

A process evaluation following the UK Medical Research Council’s process evaluation framework was conducted alongside a randomised controlled trial (RCT).

**Setting:**

The intervention was conducted in Swedish primary healthcare.

**Participants:**

Persons at high risk of stroke participating in the Make My Day, a 10-week group-based intervention (n=122) and healthcare professionals delivering the intervention (n=10).

**Methods:**

Quantitative data from logbooks, surveys and app registrations, and qualitative data from interviews, logbooks and reflexive fieldnotes were collected across five cohorts. Data were analysed separately and integrated using a convergent mixed-methods approach.

**Results:**

Fidelity varied; overall adherence to the study manual and programme content was good, while fidelity to specific session activities and session attendance varied, with a mean of 3.6 (SD 2.2) out of 6. Recruitment successfully reached individuals at high risk of stroke, but reaching a vulnerable population was challenging. Realistic, activity-focused goal setting emerged as the most perceived valued mechanism of change. Engaging everyday activities supported sustainable change when a sense of belonging was established. The group served as both a facilitator and a barrier to lifestyle change. The mobile app offered opportunities but faced usability challenges. Healthcare professionals’ delivery of the intervention was expressed as central for the change.

**Conclusions:**

This study demonstrates how fidelity, facilitation and contextual conditions shape the functioning of a complex lifestyle intervention. Supporting healthcare professionals with adequate preparation and pedagogical skills is critical to optimising the delivery and outcomes of stroke prevention interventions in primary healthcare.

**Trial registration number:**

NCT05279508.

**STRENGTHS AND LIMITATIONS OF THIS STUDY**

- A preplanned process evaluation conducted in parallel with a randomised controlled trial (RCT) study following the UK Medical Research Council’s guidance for process evaluation of complex interventions.
- The study uses a variety of quantitative and qualitative data sources collected throughout the RCT study and integrates these through a mixed-methods approach to identify facilitators and barriers to the change process.
- Data were collected from both participants and healthcare professionals, providing multiple perspectives on the intervention and its implementation.
- The researchers performing the process evaluation also conducted the RCT study, which may have introduced potential investigator bias despite the use of reflexive discussions, triangulation and consensus procedures.
- There were no observations of the healthcare professionals delivering the intervention.

## Introduction

 Stroke prevention interventions addressing lifestyle habits are strongly advocated.^1–3^ Nonetheless, there is low adherence to recommended stroke prevention guidelines in primary healthcare (PHC)^4 5^ and persons with stroke risk factors.^6^ There is a need for preventive interventions with a holistic multifactorial approach to address the complexity of lifestyle habits and risk factors.^4^ Complex interventions are commonly employed to address multiple risk factors through diverse strategies.^7^ However, implementing and evaluating a complex intervention is challenging and requires a clear understanding of implementation processes, contextual influences, mechanisms of change and delivery procedures. Detailed descriptions of the intervention and the delivery are often insufficient, resulting in a ‘black box’ that limits interpretability and replication.^8^ A process evaluation could provide essential insights into how and why a complex intervention succeeds or fails by examining implementation, context and mechanisms of change. Such evaluation is crucial for ensuring accurate interpretation of outcomes within healthcare settings.^9 10^

PHC can be at the centre of a reorganised healthcare system to address health promotion and disease prevention.^1 11 12^ A comprehensive transformation of Swedish healthcare, Good Quality Local Healthcare, is underway, with a focus on transforming PHC into the hub of the healthcare system.^12^ The reform seeks to develop more patient-centred and cohesive care tailored to individual needs. This requires ongoing education for healthcare professionals (HPs) to ensure they possess the skills to develop and enhance PHC, meeting future challenges.^13 14^ Based on this decentralised yet universally accessible PHC system, the Make My Day (MMD) intervention was developed and implemented to support stroke prevention. The MMD intervention is a multifactorial stroke prevention intervention grounded in engaging everyday activities (EEAs) augmented by m-health to facilitate the change process into health-promoting lifestyle habits, as described in detail elsewhere.^15 16^

The effect of the MMD intervention on stroke risk was evaluated in a full-scale randomised controlled trial (RCT), which demonstrated a 61% reduction in stroke risk 12 months after participation compared with controls.^16^ While the RCT established the intervention’s effectiveness, it could not explain how participants translated intervention strategies into everyday routines, which behaviour change processes supported engagement in health-promoting activities, what barriers and facilitators influenced participation and adherence, or how contextual conditions within PHC affected intervention delivery. To address these knowledge gaps, a process evaluation was conducted alongside the effectiveness study. By examining participants’ experiences and data logged during the RCT, the process evaluation provides insights into implementation quality and fidelity, mechanisms of change, and the influence of contextual factors on intervention delivery and outcomes.^17^ The aim of this study was to describe how and under what conditions the MMD intervention was delivered and experienced, in order to better understand its implementation and mechanisms of change.

## Methods

This paper will be presented and structured based on the UK Medical Research Council (MRC) guidelines components of process evaluation: implementation process and mechanisms of change incorporated with context to explore what and how the MMD intervention facilitates participants’ lifestyle change.^9 17^ The implementation process includes what was implemented and how; reach (whether the intended population was included and how); dose (the amount of intervention delivered); fidelity (whether the intervention was delivered as intended); and adaptation (any adjustments made to the intervention). For the purpose of this study, mechanisms of change are the parts in the intervention that bring about and trigger change.^8 10^ Context includes external factors that may act as facilitators or barriers to the implementation and its effects.^17^ In addition, the Promoting Action on Research Implementation in Health Services (PARIHS) framework was used to contribute to the discussion guiding the interpretation and informing the study’s implications.^18 19^ For the purpose of this study, the interplay of the PARIHS elements Context, Evidence and Facilitation was used to understand and discuss whether and in what way change was supported and sustained. The process evaluation was further informed by the logic model (figure 1), which depicts the intended causal relationships among inputs, outputs and outcomes.

**Figure 1:**
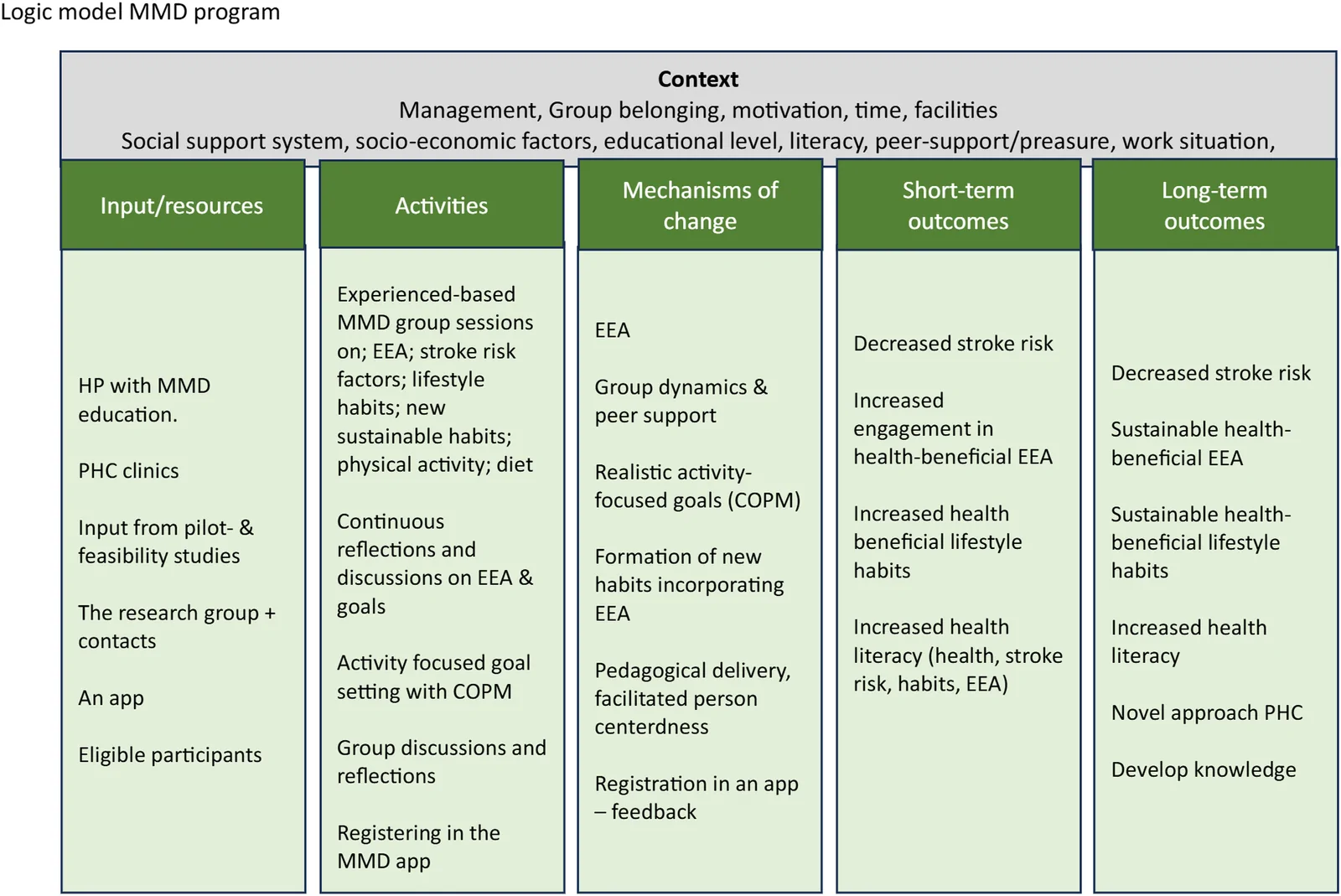
The logic model of the MMD intervention, which includes all components of process evaluation, and for the purpose of this study, the implementation process and mechanisms of change with context integrated are addressed. The logic model maps the programme theory of MMD, how, what and for whom the intervention is intended to work, illustrating causal steps and pathways between activities and intended outcomes.^9 17^ COPM, Canadian Occupational Performance Measure; EEA, engaging everyday activity; HP, healthcare professionals; MMD, Make My Day; PHC, primary healthcare.

### Study design

This process evaluation was performed alongside the RCT study using quantitative and qualitative data and methods through a convergent mixed methods approach, as recommended by the MRC guidelines.^9^ Quantitative data from logbooks, digital surveys and log data in REDCap, and app registrations were collected in parallel with qualitative data from interviews with persons at risk for stroke, HPs logbooks, and the researchers’ reflexive field notes. Quantitative and qualitative data were analysed separately and subsequently integrated to enrich the interpretation of the findings and enhance understanding of implementation process, contextual influences and mechanism of impact. Analysis from qualitative interviews with next of kin has been reported elsewhere.^20^ Due to the scope and aims of the present study interviews with HPs will be presented in a separate study.

### Setting

This study was conducted within Swedish PHC, where the MMD intervention was delivered by allied HP (occupational therapist, physiotherapist and dietician) within PHC rehabilitation units. The intervention was not part of the routine duties of HP, and before implementing the MMD intervention in the study, they had to complete an MMD education course provided by the research team. The education consisted of two face-to-face and one digital group session using a collaborative learning model complemented with their own study time during a 2-week period. Further detailed information regarding the MMD education can be found in an earlier publication by Jakobsson *et al*.^21^

### Recruitment

The recruitment of persons at risk was used in social media (Facebook) as well as advertisements in local newspapers and flyers in PHC clinics in lower socioeconomic areas (figure 2). Detailed information about the recruitment is provided elsewhere.^16^ PHC rehabilitation units were purposively recruited across the greater region of Stockholm (2021–2022), to ensure representation from different socioeconomic areas. MMD project researchers held information sessions with managers at the interested units. Each unit needed a rehabilitation team of an occupational therapist, physiotherapist and dietitian, facilities that could accommodate up to 16 participants, and allocated time for MMD education and intervention. The HP either announced their interest in joining or were allocated by their managers to join.

**Figure 2:**
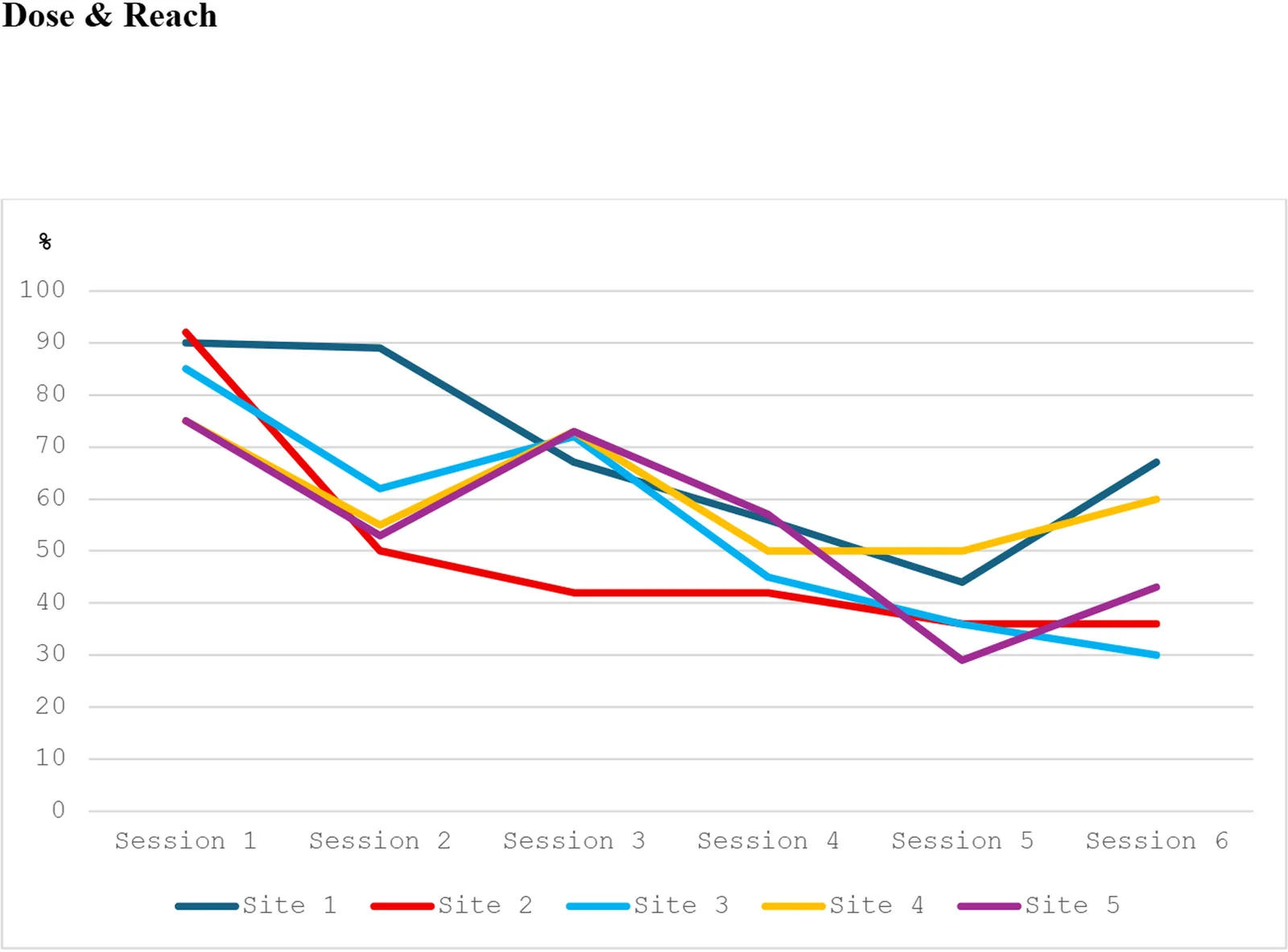
Participation in each session displayed in percentage. The number of participants included to each site differed, depicting the percentage of participants attending out of possible attendees, excluding drop-outs at each site and week/session.

### Participants

This study involved all 122 persons at high risk for stroke from the RCT participating in any of the five cohorts that received the MMD intervention. The persons at risk were randomised to either intervention (IG) or control (CG) group. All participants, IG and CG, formulated individual activity-focused goals at baseline in relation to their change process, which were evaluated at each follow-up. Furthermore, all 10 HPs from the 3 participating PHC rehabilitation units that delivered the MMD intervention programme were included (table 1).

**Table 1:** Characteristics of participants

		All participants n=122	Intervention group n=63	Interview sample n=20
Persons at risk for stroke	Female n (%)	61 (50)	31 (49.2)	10 (50)
	Age, range, m (SD)	55–75, 62 (5.2)	61.7 (4.9)	60.2 (3.5)
	Living together n (%)	76 (62)	39 (62)	11(55)
	Working n (%)	115(94)	60(95)	17(85)
	Educational level >12 years n (%)	97(80)	50(79.4)	16(80)
	European ethnicity n (%)	121(99)	62(98.4)	19(95)
	Born in Sweden n (%)	84(69)	38(64)	12(60)
Healthcare professionals	Female n (%)	8 (80)		
	Healthcare profession (n)			
	Occupational therapist	4		
	Physiotherapist	3		
	Dietician	3		
	Years in profession, range, (median)			
	Occupational therapist	8–14 (10)		
	Physiotherapist	8–28 (14)		
	Dietician	1–34 (1)		

### Patient and public involvement

Participants in MMD have previously been invited and involved in developing HP education and refining the MMD intervention. Participants will be invited to co-create further refinement to the intervention.

### The MMD intervention

The MMD intervention is a 10-week, multifactorial stroke prevention programme developed for Swedish PHC that consists of 6 themed 90-minute group sessions, each with (1) information, (2) reflection and (3) activity-based component.^16^ Participants used a mobile phone app to register goals, participation in EEAs, diet and physical activity. An interprofessional team, including an occupational therapist, physiotherapist, and dietitian, led by the occupational therapist, collaborated to create a supportive environment to enable persons at risk of stroke to pursue a healthy lifestyle. EEA, aligning the six group sessions, fosters joy, motivation, participation, and supports sustainable lifestyle changes.^22^ The MMD intervention incorporates cornerstone strategies designed to facilitate participants’ change processes illustrated in figure 3. The development of the MMD intervention has been ongoing with feasibility and pilot study proceeding the RCT study.^23–25^ The results of the MMD RCT study have been reported in detail elsewhere.^16^

**Figure 3:**
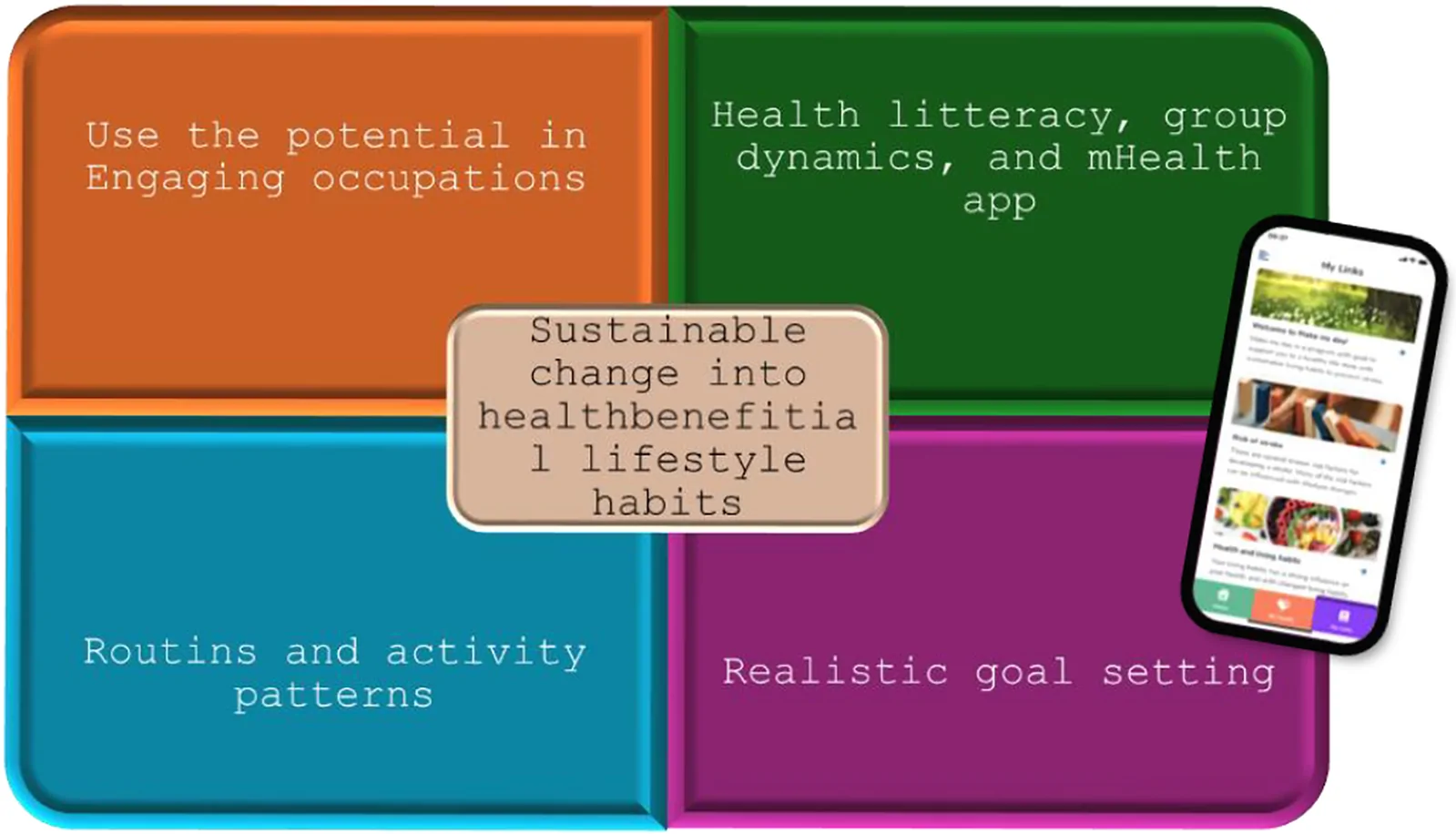
The cornerstone strategies to facilitate the change process in the MMD intervention programme—the intended mechanisms of change. (1)Participation in health-promoting EEA; (2) setting realistic, activity-focused goals that promote new habits^44^; (3) mHealth and (4) the group dynamics. The strategies build on evidence such as behaviour change techniques,^45^ engaging everyday activities,^25 46^ and national and international guidelines on CVD and stroke risks and addressing (un)healthy lifestyle habits.^11 14^ CVD, cardiovascular disease; EEA, engaging everyday activity; MMD, Make My Day.

### Data collection

Data were gathered from all participants at baseline, postintervention and 12 months, between 2022 and 2024. Data were collected from multiple sources from all participants in the five cohorts and HP as well as from selected samples, as recommended by Moore *et al*.^17^ Additionally, the data collectors, including the first author (CJ), a postdoctoral researcher, and two research assistants, wrote reflexive field notes after each data measurement meeting.

The MMD app, used by the IG as part of the intervention, was used to register daily data and provide the participant with input on their goal fulfilment, participation in EEA, activity patterns and dietary intake over the 10 weeks of the intervention. These data were used to inform on app use.

Four interviewers, including CJ, conducted all qualitative interviews with a purposive sample from the IG (n=20). After completing the follow-up, all IG participants were informed and invited to take part in follow-up interviews. To achieve a representative selection from the IG, 40% of completed follow-ups across the five cohorts were interviewed. The first four participants from each of the five cohorts who accepted were selected in the order of acceptance, maintaining gender balance.^17^ All interviews used a semistructured format based on the logic model, ensuring consistent questions and qualitative insights. Interviews focused on experiences with MMD and its potential impact on change processes and lifestyle change after 12 months (see the interview guide in the online supplemental file. The open questions covered the IG participants’ experience with MMD, performing lifestyle changes, the group’s influence and perceived barriers or facilitators. Each interview lasted 35–60 min, was recorded, and included verbatim transcripts and reflective notes.

The logbooks kept by the HP during the intervention entailed questions on dose, fidelity, adaptation and reflections. Figure 2 shows the timeline for all data collection.

### Data analysis

To assess the extent to which MMD delivery was implemented as intended, procedures and data were compared with the study protocol,^15^ programme manual and MMD logic model (figure 1). The components of process evaluation; context integrated with implementation and mechanisms of change guided the analysis.

### Quantitative data analysis

Descriptive statistics in SPSS were used to analyse and process quantitative data on baseline characteristics, dose, reach, fidelity and adaptation and mechanisms of change which are outlined in table 2.

**Table 2:** Timeline of the data collection and various types of data collected and data analysis process

	Recruitment	Baseline	3 months	12 months	Analysis	
**Reach**						
Quantitative data	REDCap log data by researchers	–	–	–	Descriptive statistics	Synthesis of qual and quant analysis
Qualitative data	Reflexive fieldnotes by researcher, all participants	–	–	Semistructured interviews–IG n=20	Qualitative content analysis	
Dose						
Quantitative data	–	REDCap log data by researchers and response rates to instruments, all participants Logbooks by HP during the MMD sessions			Descriptive statistics	Synthesis of qual and quant analysis
Qualitative data	–	Logbooks by HP during the MMD sessions			Qualitative content analysis	
Fidelity and adaptation						
Quantitative data	–	Logbooks by HP during the MMD sessions			Descriptive statistics	Synthesis of qual and quant analysis
Qualitative data	–	Logbooks by HP during the MMD sessions	Logbooks by HP during the MMD sessions Evaluation survey of the MMD sessions–IG n=41	Logbooks by HP during the MMD sessions Semistructured interviews–IG n=20	Qualitative content analysis	
Mechanisms of change						
Quantitative data	–	–	Evaluation survey of the MMD sessions–IG n=41 MMD App-log data–IG n=63 Usability survey* of the MMD app–IG n=41	–	Descriptive statistics	Synthesis of qual and quant analysis
Qualitative data	–	Reflexive fieldnotes by researchers at measurement session, all participants	Evaluation survey of the MMD sessions–IG n=41 Reflexive fieldnotes by researchers at measurement session, all participants	Semistructured interviews–IG n=20 Reflexive fieldnotes by researchers at measurement session, all participants	Qualitative content analysis	

The data collection timeline was consistent across all five overlapping cohorts. The analysis of the quantitative and qualitative data was initially performed separately, followed by a merging and synthesis of the results with a convergent focus. The illustration is from the thesis of Johnsson.

* The survey was inspired by the System Usability Scale (Brooks, 1986), from 0 to 100 and was used to capture the IG level of satisfaction with the MMD app. The surveys entail both preset answers and free-text questions.

HP, healthcare professional; IG, intervention group; MMD, Make My Day.

### Qualitative data analysis

Qualitative content analysis^26^ was used to analyse interviews, logbooks and reflexive fieldnotes, seen in table 2. CJ performed the analysis structured with Atlas.ti. A deductive approach, using an analytical framework based on the process evaluation components, context integrated with implementation and mechanisms of change, alongside the activities in the logic model, guided the analysis^17^

### Synthesis

Integration of quantitative and qualitative analyses occurred at the interpretation stage using an integration matrix that juxtaposed quantitative findings with qualitative themes, consistent with a convergent mixed-methods approach.^27^ Findings were compared with identify convergence, complementarity and divergence.

Qualitative findings were used to provide contextual understanding of the quantitative results and potential mechanisms underlying observed patterns. Similarly, quantitative findings were used to assess the extent to which qualitative themes were reflected across the broader participant sample. Discrepancies were explored through revisiting both datasets to identify possible explanations related to individual variation, contextual influences, or differences in intervention experiences.

The final synthesis was developed through an iterative process in which findings from both datasets were compared, discussed, and interpreted collectively by the research team.

### Reflexivity

To enhance trustworthiness and minimise potential investigator bias, several strategies were employed throughout the study. The data collectors, including the first author (CJ), a postdoctoral researcher, and two research assistants, had continuous reflexive discussions and consensus meetings. The research team conducted regular reflexive discussions to critically examine assumptions, interpretations, and the potential influence of researchers’ preunderstanding on data collection and analysis. Only the first author (CJ), was involved in both data collection and analysis. Coding and analytical decisions, initially performed by CJ and A-HP, were discussed among all authors until consensus was reached. Findings were continuously compared across data sources, thereby strengthening the credibility and trustworthiness of the results.

## Results

The results are presented in a structure of MRC process evaluation components integrating quantitative and qualitative data. The analysis of the experiences of the IG formed an overarching category: the intricate mosaic of (performing sustainable) lifestyle changes, which entailed five subcategories described in detail under the paragraph Mechanisms of change.

### The implementation of MMD

#### Reach

Figure 4 shows the recruitment of persons at risk of stroke, which was conducted in five phases. Phase 2 was initiated when PHC clinics for cohorts three and four had been enlisted. Analysis showed an over-representation of women and difficulty reaching low socioeconomic and non-Swedish-born groups, despite outreach through clinics and local newspaper advertisements. Consequently, the social media campaign was adjusted in phase 5 to target men and underserved populations, achieving gender balance. However, recruitment from low socioeconomic areas and of non-Swedish born remained limited. A total of 122 participants with ≥3 high stroke risk factors, including ≥2 modifiable ones, were enrolled (figure 4).

**Figure 4:**
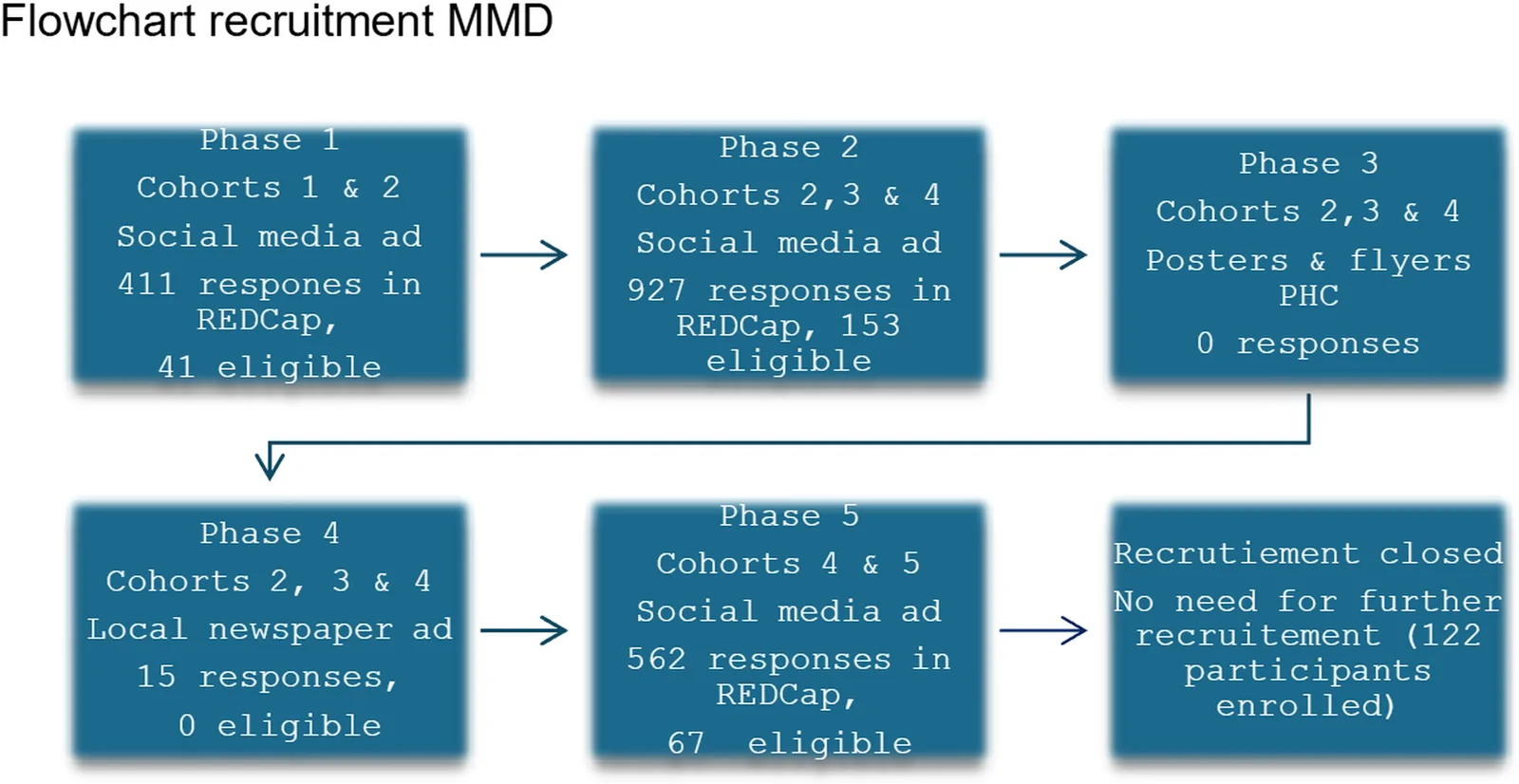
Flow chart over the MMD recruitment process and strategies of persons at risk for stroke and the PHC units for each cohort. MMD, Make My Day; PHC, primary healthcare.

Persons at risk for stroke reported encountering the study via social media and expressed that it resonated with their personal circumstances, particularly in relation to a progressive decline in lifestyle habits. For others, it was described as an alarm igniting reflection about their stroke risks and fear of having a stroke, motivating a change. All expressed awareness of the need for changes but struggled to initiate or maintain lifestyle changes on their own.

When I heard about this, I read about this study, I applied. And was so happy when I got included. Because I feel like I can’t do it on my own Participant 6, site 4

Of the 122 enrolled participants, the drop-out rate for postintervention follow-up was 16 (13%) and an additional 3 (2.5%) for the 12-month follow-up. The total dropout rate was 15.6%, which is within the accepted retention rate of <20%.^28^ However, the decline in participation during the sessions was below the recommended threshold of ≥75% of the sessions completed by ≥70%^28^ illustrated in figure 2.

#### Dose

Dose delivered was high: the HP delivered all six planned sessions (100%). Dose received was assessed as attendance at each of the six MMD group sessions and is presented in percentage of allocated participants attending per session at each site (figure 2). The mean participation rate in each session across all five sites combined was 52.8%, and when calculated on participants who completed the programme, it was 62.8%. Overall, allocated participants attended a mean of 3.6 (SD 2.2) of the six group sessions. Among participants who completed the programme (n=53), the mean number of sessions attended was 4.4 (SD 2.1) out of 6. Reasons given for absence included lack of time due to work, illness or poor weather conditions. However, most did not report their absence, so the exact reasons remain unknown.

### Fidelity and adaptation

To assess intervention fidelity, the study protocol, logic model, programme manual, HP logbooks, interviews with IG and researchers’ reflection notes were compared. HP education, assessments and session content were delivered as planned. However, some activities (ie, the EEA interview performed in session 1) were not fully implemented in the sessions. Time constraints led to activities being cut short or rushed discussions, which left the HP with feelings of discomfort of not delivering.

It was difficult to keep the time as discussions ran long och the grouped asked a lot of questions och discussed the answers – HP site 1

The persons at risk for stroke noticed time constraints and observed that parts of the agenda were not followed. They expressed regret over missing the opportunity to discuss their goals or EEA with other participants. They identified this as part of sessions that could advance their change process. Many described the discussions as highly valuable. Some expressed a desire to prolong the discussions, while others noted that a large amount of time was spent getting stuck on issues less relevant to the whole group and that the speaking space was not facilitated among the participants.

There was a time when it felt a little too forced. You know that if you are a group that likes to discuss it, it can be good that you are allowed to do so, because I think that when you notice that others have the same problems and some may have a smart solution to the problems that you have, it gives a lot of added value Participant 3 Site 2

Adaptations of intervention components, such as the planned activities carried out during the sessions, varied by site due to restrictions in the facilities at hand

The food lab there were neither time nor space in the facilities for that. I had prepared a fruit salad before the session and a follow-up discussion – HP Site 3

The participants’ varying prerequisites or weather conditions sometimes affected the choice or implementation of the activity. Participants noticed that some aspects did not go as planned and felt that they missed out on shared experiences. Many spoke highly of the joint activity for enhancing the group and process.

Persons chose to not do the Walk and Talk due to slippery walkways. One person was not able to walk longer distances – HP Site 4

### Mechanism of change

In evaluation surveys, persons at risk of stroke described the group as their primary facilitator for change, followed by realistic goals, EEA and meaningful discussions on these topics. Motivated HPs positively influenced change, and variation offered in activities served as eye-openers, supporting increased health literacy. However, barriers included the app, delivery, diverse groups, and declining participation. Group dynamics were found to either facilitate or hinder change, depending on context. In the interviews, the persons at risk for stroke describe and elaborate on the mechanisms in their change process. An overarching category, the intricate mosaic of performing lifestyle changes was identified entailing five subcategories: (a) the group, a facilitator and a barrier, (b) the benefit of realistic perspectives in formulating goals, (c) the multilayered and complex nature of engaging in everyday activities, (d) the app, lost possibilities and (e) the focal role of intervention communication and interaction.

### The intricate mosaic of performing lifestyle changes

Participants described becoming more aware of their risk factors and current lifestyle habits, while also acknowledging the challenges of adopting new health-promoting behaviours and applying new knowledge in their everyday life.

I didn’t even think about being in a stroke risk group, because it’s terrible. So, I’ve got that with me, so to speak. And I have to think about this with my diet and exercise. Above all. Yes. For example, now that the weather is nice, I take the bike, go for a ride, and buy flowers. Because it’s also fun. Or meet a friend, that I, who I cycle to Participant 2, site 1

They also reflected on how, to an extent, they already knew about lifestyle risk factors and health, but that it had been hard to incorporate new habits into their everyday lives. Others reflected on the challenge of maintaining change and not falling back into old patterns before the change one is trying to perform takes hold.

No, not really. It’s the kind of stuff you know about but don’t do, but it’s always a kick in the bum to do it better. You should eat healthy things, more fish, more vegetables, and exercise, of course. That’s how it is. Like, I’m not as afraid to park the car a bit further away and walk a distance, so you think about it a little more, I think Participant 1, site 3

### The group: a facilitator and a barrier

Participants expressed mixed feelings about the group. Most regarded it as a positive aspect of the programme, emphasising the sense of connection and understanding it fostered. The group served as a safe haven for sharing experiences and struggles that they usually kept to themselves, offering support and advice without judgement. Participants expressed feeling as one of many, not the only one, who do things wrong or fail.

I felt nervous at first but once you saw that we all had struggles and experiences and that no one was one of those who does everything right. It was a relief and we shared openly but with a lot of humor which was a wonderful way to talk about serious things that we were doing Participant 3 site 5

The group’s commitment and trust were challenged by declining numbers, prompting participants to reflect. Some wondered whether absentees or their families faced illness or difficulties or felt unready for change. Some expressed how this negatively impacted group dynamics by reducing discussion and reflection, possibly limiting dialogue. Some also noted trust issues, which made them hesitant to share personal thoughts with others who might have been absent.

But to create a group feeling, you need to have a better continuity with those who are in the group. Somehow, so if someone comes in who hasn’t been there for a while and then it becomes like a new group in some way. You kind of become. Strangers each time Participant 2, site 2

### The benefit of realistic perspectives in formulating goals

Participants reflected on their goals, noting that setting goals relevant to their current circumstances and interests was essential. Some participants expressed that reflection and discussions during the goal-setting process helped them formulate realistic goals, which supported their change process.

So already when I was included to the program, I was incredibly excited and motivated and I changed my lifestyle quite dramatically and lived according to the goals that I set up at this first interview or meeting and what it is was strengthened during the program and continued for a few months afterwards as well. Participant 5, site 4

For many, the goals became an integrated part of everyday life. While others kept reminders or viewed the goals as agreements with themselves to stay on track.

The goals are still there, although I don’t think of them as goals, I just think that this is what I’m going to work with. I’ve already passed these goals, but I still hang on to them, because they’re still good goals. It becomes like a reminder Participant 6, site 4

### The multilayered and complex nature of engaging everyday activities

Engaging everyday activities (EEAs) was a new concept for the participants, and they described their perception of the familiarisation process differently. Many participants described reassessing an existing activity or interest and finding it engaging and valuable for their well-being and health. For some, a new activity, such as cooking, became an engaging activity and part of their lifestyle. Others reflected on an already existing activity, in which taking the bike to see a friend instead of taking the bus gained a new, deeper meaning for well-being.

And it was a bit more fun to cook the vegetarian food than to cook a piece of meat. It became more fun to cook, more fun to eat, I think. Exactly, it has probably gone from necessity to engaging activity, actually Participant 7, site 1

For others, activities shared with others, such as feeling a sense of connectedness or togetherness through a relationship with a group, community or context, triggered engagement and transformed the activity, such as singing in a choir or spending time with grandchildren, into an engaging activity.

Yes, yes exactly, well, both bridge and choir are so engaging that you forget to eat, I will say. The choir is so much more than just going there and singing, it’s a togetherness and a belonging, you feel better when you’re there Participant 1, site 4

There were also examples of participants who objected to a general idea that EEAs was solely beneficial for health. This limited the scope of the discussions and reflections, as it was perceived that certain interests and habits weren’t open for discussion as engaging activities.

Yes, well, what engages me is this, right, and that’s completely wrong, but if I really got to choose, I would just lie on the couch [laughs]. We would drink some wine or coffee and then I would listen to music and maybe dance a little. Participant 3, site 1

Some struggled to understand EEAs, finding sessions lacking explanations or examples. Discussions were at times described as unclear, with HP having difficulty conveying meaning and context.

What you feel is engaging and so on. And there was a bit of conceptual confusion, what that meant. Some didn’t understand at all and some understood more and so on. But it was a discussion Participant 8, site 3

### The app—lost possibilities

Participants used the app in various ways during the 10-week programme, as indicated by their frequency of use, activity types and days logged. App registration was low at the group level, peaking at 3.2 days per week in the second week, then steadily declining to 1 day per week in weeks nine and ten. The fifth and final site had the highest average registration, at 3.3 days per week, while sites 2 and 4 had the lowest, at 1.0 and 0.8 days per week, respectively. Several technical issues were reported during app use, particularly during the first cohorts, which influenced participants’ perceptions and engagement. The participants rated the usability of the app, which serves as a combined measure of effectiveness, efficacy and satisfaction, m (SD) 53.9 (2.88), range: 20/97.5.

Many participants saw the benefits of an app in the intervention for feedback and motivation. Several had tried or started using different devices or apps with positive results.

I thought that was fantastic and it ended up being yes, but OK then I bought one of these (pointing to the watch on the arm) just to be able to have one myself……, well yes, but to at least have an idea. Participant 4, site 5

However, the app was often described as malfunctioning or too complicated and time-consuming to use. Many grew tired of the app and gradually stopped using it.

Well, the technology didn’t always work, it wasn’t user-friendly. Ehm but I know that this was something that the group also experienced as very disturbing that the technology didn’t work. Participant 1, site 1

### The focal role of intervention communication and interaction

An unanticipated mechanism of change identified was the importance of the HP and their role in conveying the intervention to participants, becoming both facilitators and barriers in the participants’ change process. The persons at risk of stroke perceived and described the HP’s delivery differently. Some participants regarded the interaction as motivating and encouraging without passing judgement, which supported the change process. Others described difficulties among HP in guiding the group through the sessions, as they permitted too much latitude for participants to set the agenda. Additionally, some expressed a need for more prepared HP, equipped with in-depth knowledge of the topics and concepts being presented and discussed, and a desire for HP to convey engagement and motivation.

I felt that those who, ehm, were in charge, did not always choose to steer the group so much. Uhm, which contributed to leaving an open window for the group to opt out of certain things in this eh activity schedule. Participant 6, site 2

This result showed the importance of the HP as a facilitator for the group dynamic and the persons’ change process.

## Discussion

The aim of this study was to analyse and evaluate the implementation process and the mechanisms of change of the MMD. The results demonstrate how fidelity to the MMD intervention and adaptations in delivery varied between sites and cohorts, and how deviations from fidelity negatively impacted the participants’ experienced change process. It also illustrates the decline in attendance rate. Setting realistic activity-focused goals was the most valued mechanism of change by the participants, with many having incorporated their goals into their everyday life. The results highlight the dualistic nature of the mechanisms of change, acting as both facilitators and barriers to the participants’ change processes. An unanticipated perceived mechanism of change, the focal role of intervention communication and interaction in the participants’ change process, was identified during the analysis. This discussion is structured around facilitation mechanisms underpinned by context and evidence that guide and support confidence, reflection and change strategies, as well as create environments and tools to navigate challenges.^18 19^

### The most valued mechanisms of change

Participants described setting realistic activity-based goals as a major facilitator, as it built on their current circumstances and interests. Goal-setting has been shown to enhance self-awareness by encouraging individuals to articulate their thoughts on meaningful activities, which subsequently boosts motivation and commitment to participate in the intervention.^29^ A scoping review of the Lifestyle Redesign studies demonstrated how reflection and discussions during the goal-setting process facilitated insights, igniting the participants’ change process.^30^ Despite this, studies using lifestyle interventions seldom describe, or provide reflection about, goal-setting as a behaviour change strategy.^7 31 32^ The MMD RCT study,^16^ in which both IG and CG set individual goals, shows that both groups significantly improved goal fulfilment at 12 months with no significant difference between the IG and CG and that the CG also made within-group improvements in stroke risk factors at 12 months. Together, the RCT and the current study show that setting realistic activity-focused goals is an important mechanism for lifestyle change identified by the participants.

The participants’ descriptions of the group as a nurturing environment for sharing experiences and struggles, fostering discussion and serving as a sounding board for discovering new ways of reasoning and acting that supported their change process align with the literature.^33^ Being in a place among peers without judgement or blame, facing similar challenges, the group provided a safe space for the participants.^34^ Facilitating structures for relationships through dialogue and reflexivity, which define roles and responsibilities, have been identified as a foundation for constructive collaboration and building equity.^35^ However, some participants raised concerns about the group’s diversity and how some were allowed to dominate discussions. A process evaluation found that even though lifestyle interventions can be effective in facilitating change, it also places a burden on participants, highlighting a need for increased support and resources during delivery.^36^ Ineffective intervention facilitation or unclear group structure may lead to confusion or disengagement, emphasising the importance of skilled facilitators and careful group design.^33^

Conclusions on what influenced positive results in the RCT depend on intervention fidelity^10 17^ where poor fidelity can mislead results by discarding effective or using ineffective interventions.^37^ A decline in participation across all sites, including app use, was observed, and understanding why participation declined is key to improving the intervention and interpreting RCT results. HPs cited time constraints and reluctance to interrupt discussions as reasons for omitting activities, highlighting the importance of recognising the value of joint experiences and the HPs’ role in change. The process evaluation of Aerts *et al*^5^ identified the need to upskill competence in the HP group and supportive frameworks and organisational systems as possible facilitators. Although many participants described HPs as motivating and tolerant aligning with the MMD approach creating a safe space for lifestyle change some reported perceived insecurity in the HP delivery. The MMD intervention was new to the HP who described the intervention as a novel approach.^38^ Combined, this may be related to whether HP perceived ownership of the material, consequently affecting their motivation^39^ impacting the change process.^40^ Collaborative workshops with HPs and participants led to cocreating refined HP education emphasising core concepts and pedagogical methods.^38^ Overall, these findings show the need to refine the MMD intervention and HP education to improve pedagogical delivery.

### The multilayered EEAs and the change process

EEA, identified by some HP and participants as hard to grasp, is a major concern, as it is a core concept and believed to be the key facilitator for change in MMD. This highlights the complexity of EEA and underscores the need to clarify its theoretical foundation to use its full potential. Moreover, it shows the need for adjustments and strategies within MMD to make EEA more accessible and relatable. The participants who described a sense of connectedness, togetherness, a belonging to their EEA continued to engage in these activities and incorporated them into their everyday lives. The expressed feeling of being part of a community or camaraderie, being connected with others within a shared context of interests, values, or beliefs, was key to making the activity engaging and thereby facilitating a sustainable change process. This aligns with the concept of belonging described as feeling connected to others, places, cultures, and communities, providing the context for various activities where relationships are essential to experiencing belonging.^41 42^ EEA have been found to entail a complexity in which contextual norms and values label an activity as unhealthy or immoral, instilling a sense of shame and guilt in the person, which risks omitting mention of the activity.^43^ These are important findings which have clinical implications, as an awareness of what truly engages a person and how this motivates sustainable change may help HP support individuals in addressing lifestyle changes.

### Methodological considerations

A strength of this preplanned process evaluation is its use of guidelines for performing process evaluation^17^ employing a mixed-methods approach that uses both qualitative and quantitative data, thereby providing more comprehensive and multifaceted data. The focus on evidence, context and facilitators and their dynamic interplay is a strength^18 19^ in this study to understand how change is supported and sustained. Moreover, it is a strength that data have been collected from all five sites using a consistent structure (logbooks, surveys, and interviews), collected at different time points, across the entire sample, offering a broad overview, while a selected sample provides in-depth insights.

The observed decline in participant attendance across all sites may undermine the strength of the MMD intervention, discussed in-depth in the RCT study,^16^ its motivational impact and implementation fidelity. Nonetheless, the IG exhibited a significant reduction in stroke risk, suggesting that, for some participants, minimal exposure to the intervention might have been sufficient to initiate a change process. However, as attendance data were not available at the individual level, an exposure-response analysis could not be performed, and the assumption needs further investigation. Conversely, this also prompts consideration of whether the low attendance rate could have affected the intervention’s effects and the interpretation of underlying mechanisms of change. Attendance rates were measured only at the group level, not at the individual level; this limitation precludes more detailed analysis, representing a constraint of the study.

There were no separate research teams for the effect study and this study, meaning data collection and analysis happened simultaneously. This might be a limitation because analysts were not blinded, possibly causing bias or conflicts of interest. However, the research team unity also provided benefits by sharing data collection, which lessened the measurement burden on participants. Although doing this study alongside the RCT and its analysis could risk bias, it allowed the application of this study’s results to the RCT findings, leading to effective conclusions.

We (the researchers) did not observe the HP delivering the MMD intervention; instead, we relied on their self-reported delivery recorded in a logbook and feedback from participants in surveys and interviews. This approach could potentially overlook mechanisms or factors underlying the reported results of the MMD intervention and the fidelity of its implementation. For this study, we did not conduct interviews with any participants in the CG. It would have been valuable to gain more insight into their change process. This is especially since the group showed significant within-group changes at 12 months compared with baseline, which has been discussed in detail in the RCT study.^16^ Another limitation is the homogeneous study population, which mainly consisted of European-born participants who had completed higher education. Recruiting participants from lower socioeconomic contexts proved challenging. This may limit the study’s transferability to a broader population.

## Conclusion

This process evaluation provides important insights into the mechanisms and contextual factors influencing lifestyle change within the MMD intervention. Together with the RCT findings, the results support the potential of MMD as a feasible health-promoting intervention in PHC for facilitating sustainable lifestyle change and reducing stroke risk. The clinical implications highlight goal setting and EEA as key intervention components that may support behaviour change by supporting individuals to identify meaningful activities and achievable means to healthier lifestyles. The results also underscore the importance of carefully considering group composition and participant inclusion, as group processes may function as both facilitators and barriers to change. Furthermore, successful implementation appears to require adequate preparation, training and support for HP to strengthen confidence, intervention fidelity and perceived credibility. Collectively, these findings have informed refinements to both the HP education and the MMD intervention and provide practical guidance for future implementation and adaptation in PHC across a broader range of populations with preventive health needs. The findings have also guided the further development of both the HP education and the MMD intervention, including applications related to return to work and vulnerable populations.

## Supplementary material

10.1136/bmjopen-2026-123012 online supplemental file 1

## Data Availability

Data are available on reasonable request.

## References

[1] World Health Organization (2025). Global ncd compact 2020-2030. https://www.who.int/initiatives/global-noncommunicable-diseases-compact-2020-2030.

[2] Yusuf S, Joseph P, Rangarajan S (2020). Modifiable risk factors, cardiovascular disease, and mortality in 155 722 individuals from 21 high-income, middle-income, and low-income countries (PURE): a prospective cohort study. Lancet.

[3] Wu J, Feng Y, Zhao Y (2024). Lifestyle behaviors and risk of cardiovascular disease and prognosis among individuals with cardiovascular disease: a systematic review and meta-analysis of 71 prospective cohort studies. Int J Behav Nutr Phys Act.

[4] Kotseva K, De Backer G, De Bacquer D (2021). Primary prevention efforts are poorly developed in people at high cardiovascular risk: A report from the European Society of Cardiology EURObservational Research Programme EUROASPIRE V survey in 16 European countries. Eur J Prev Cardiol.

[5] Aerts N, Van Royen K, Van Bogaert P (2023). Understanding factors affecting implementation success and sustainability of a comprehensive prevention program for cardiovascular disease in primary health care: a qualitative process evaluation study combining RE-AIM and CFIR. Prim Health Care Res Dev.

[6] Kotseva K, De Backer G, De Bacquer D (2019). Lifestyle and impact on cardiovascular risk factor control in coronary patients across 27 countries: Results from the European Society of Cardiology ESC-EORP EUROASPIRE V registry. Eur J Prev Cardiolog.

[7] Lontano A, Marziali E, Galletti C (2022). A real opportunity to modify cardiovascular risk through primary care and prevention: A pilot study. Front Public Health.

[8] Saarijärvi M, Wallin L, Bratt E-L (2020). Process evaluation of complex cardiovascular interventions: How to interpret the results of my trial?. Eur J Cardiovasc Nurs.

[9] Skivington K, Matthews L, Simpson SA (2021). A new framework for developing and evaluating complex interventions: update of Medical Research Council guidance. BMJ.

[10] Moore GF, Evans RE, Hawkins J (2019). From complex social interventions to interventions in complex social systems: Future directions and unresolved questions for intervention development and evaluation. Evaluation (Lond).

[11] Bushnell C, Kernan WN, Sharrief AZ (2024). 2024 Guideline for the Primary Prevention of Stroke: A Guideline From the American Heart Association/American Stroke Association. Stroke.

[12] Swedish National Board of Health and Welfare (2021). Good quality, local healthcare - a primary care reform. https://regeringen.se/contentassets/3fcc1ab1b26b47bb9580c94c63456b1d/god-och-nara-vard-sou-2019-29.pdf.

[13] Swedish National Board of Health and Welfare (2025). Uppföljning av omställningen till en god och nära vård. www.socialstyrelsen.se.

[14] The Swedish National Board of Health and Welfare (2024). National guidelines for prevention and treatment of unhealthy lifestyle habits. https://www.socialstyrelsen.se/globalassets/sharepoint-dokument/artikelkatalog/nationella-riktlinjer/2024-11-9272.pdf.

[15] Patomella A-H, Guidetti S, Hagströmer M (2023). Make My Day: primary prevention of stroke using engaging everyday activities as a mediator of sustainable health - a randomised controlled trial and process evaluation protocol. BMJ Open.

[16] Johnsson C, Asaba E, Guidetti S (2025). Make My Day - Stroke Prevention Grounded in Engaging Everyday Activities in Primary Healthcare - A Single-Blinded Randomised Controlled Trial. J Prim Care Community Health.

[17] Moore GF, Audrey S, Barker M (2015). Process evaluation of complex interventions: Medical Research Council guidance. BMJ.

[18] Kitson AL, Rycroft-Malone J, Harvey G (2008). Evaluating the successful implementation of evidence into practice using the PARiHS framework: theoretical and practical challenges. Implement Sci.

[19] Bergström A, Ehrenberg A, Eldh AC (2020). The use of the PARIHS framework in implementation research and practice-a citation analysis of the literature. Implement Sci.

[20] Norefors K, Asaba E, Åkesson E (2026). Perceptions of Family Support in the Stroke Prevention Program Make My Day. Can J Occup Ther.

[21] Jakobsson E, Johnsson C, Schimmer R (2025). Co-designing interprofessional education in primary healthcare: an illustration from the Make My Day stroke prevention project. J Interprof Care.

[22] Asaba E, Bergström A, Patomella A-H (2022). Engaging occupations among persons at risk for stroke: A health paradox. Scand J Occup Ther.

[23] Patomella A-H, Mickols G, Asaba E (2018). General practitioners’ reasoning on risk screening and primary prevention of stroke - a focus group study. BMC Fam Pract.

[24] Patomella A-H, Farias L, Eriksson C (2021). Engagement in Everyday Activities for Prevention of Stroke: Feasibility of an mHealth-Supported Program for People with TIA. Health Care (Don Mills).

[25] Mälstam E, Asaba E, Åkesson E (2023). The Feasibility of Make My Day-A Randomized Controlled Pilot Trial of a Stroke Prevention Program in Primary Healthcare. Int J Environ Res Public Health.

[26] Lindgren BM, Lundman B, Graneheim UH (2020). Abstraction and interpretation during the qualitative content analysis process. Int J Nurs Stud.

[27] Creswell JW, Plano Clark VL (2017). Designing and Conducting Mixed Methods Research.

[28] Pfledderer CD, von Klinggraeff L, Burkart S (2024). Consolidated guidance for behavioral intervention pilot and feasibility studies. Pilot Feasibility Stud.

[29] Larsen AE, Law M (2025). Canadian Occupational Performance Measure the state of the art - a review. Scand J Occup Ther.

[30] Pyatak EA, Carandang K, Rice Collins C (2022). Optimizing Occupations, Habits, and Routines for Health and Well-Being With Lifestyle Redesign®: A Synthesis and Scoping Review. Am J Occup Ther.

[31] Lönnberg L, Ekblom-Bak E, Damberg M (2020). Reduced 10-year risk of developing cardiovascular disease after participating in a lifestyle programme in primary care. Ups J Med Sci.

[32] Sisti LG, Dajko M, Campanella P (2018). The effect of multifactorial lifestyle interventions on cardiovascular risk factors: a systematic review and meta-analysis of trials conducted in the general population and high risk groups. Prev Med.

[33] Borek AJ, Abraham C (2018). How do Small Groups Promote Behaviour Change? An Integrative Conceptual Review of Explanatory Mechanisms. Appl Psychol Health Well Being.

[34] McGill B, Corbett L, Grunseit AC (2022). Co-Produce, Co-Design, Co-Create, or Co-Construct—Who Does It and How Is It Done in Chronic Disease Prevention? A Scoping Review. Health Care (Don Mills).

[35] Pérez Jolles M, Willging CE, Stadnick NA (2022). Understanding implementation research collaborations from a co-creation lens: Recommendations for a path forward. Front Health Serv.

[36] Jayaprakash M, Puri-Taneja A, Kandula NR (2016). Qualitative Process Evaluation of a Community-Based Culturally Tailored Lifestyle Intervention for Underserved South Asians. Health Promot Pract.

[37] Ginsburg LR, Hoben M, Easterbrook A (2021). Fidelity is not easy! Challenges and guidelines for assessing fidelity in complex interventions. Trials.

[38] Johnsson C, Jakobsson E, Hagströmer M (2025). Refining the Make My Day stroke prevention intervention for primary healthcare through co-creation with stakeholders. Res Involv Engagem.

[39] Grindell C, Coates E, Croot L (2022). The use of co-production, co-design and co-creation to mobilise knowledge in the management of health conditions: a systematic review. BMC Health Serv Res.

[40] Constantin N, Edward H, Ng H (2022). The use of co-design in developing physical activity interventions for older adults: a scoping review. BMC Geriatr.

[41] Wilcock A, Hocking C (2015). An Occupational Perspective of Health.

[42] Hitch D, Pépin G, Stagnitti K (2014). In the footsteps of Wilcock, Part two: The interdependent nature of doing, being, becoming, and belonging. Occup Ther Health Care.

[43] Johnsson C, Farias L, Patomella A-H (2026). Isn’t that also good for my health? The shifting nature of sanctioned and non-sanctioned occupations in the context of lifestyle change. J Occup Sci.

[44] Law M (2020). COPM Canadian Occupational Performance Measure: Svensk Version.

[45] Michie S, Richardson M, Johnston M (2013). The behavior change technique taxonomy (v1) of 93 hierarchically clustered techniques: building an international consensus for the reporting of behavior change interventions. Ann Behav Med.

[46] Jonsson H (2008). A new direction in the conceptualization and categorization of occupation. J Occup Sci.

[47] Johnsson C (2025). Complex paths of lifestyle change in stroke prevention: unveiling seeds and processes of engaging occupations. Karolinska Institutet.

