# Supplementary material for: Performing lifestyle changes and reducing stroke risk: a process evaluation of a stroke prevention intervention
Source: BMJ Open. 2026 Sep 30;16(9):e123012. doi: 10.1136/bmjopen-2026-123012 (PMC13629870; doi:10.1136/bmjopen-2026-123012)
Supplement: Supplementary file 1 [file bmjopen-2026-123012.reviewer_comments.pdf]

PEER REVIEW HISTORY

BMJ Open publishes all reviews undertaken for accepted manuscripts. Reviewers are asked to complete a checklist review form and are provided with free text boxes to elaborate on their assessment. These free text comments are reproduced below.

ARTICLE DETAILS

Title (Provisional)

Performing lifestyle changes and reducing stroke risk -A process evaluation of a stroke prevention intervention

Authors

Johnsson, Cecilia; Asaba, Eric; Guidetti, Susanne; Hagströmer, Maria; Patomella, Ann-Helen

---

VERSION 1 - REVIEW

**Reviewer**	**1**
**Name**	**Zhu, Luwen**
**Affiliation**	**Heilongjiang University of Chinese Medicine, Fourth Affiliated Hospital**
**Date**	**08-Jun-2026**
**COI**	**None**

This study conducts a process evaluation of the Make My Day (MMD) stroke primary prevention intervention. This topic holds value for public health and primary care practice, particularly in terms of its practical significance for elucidating the mechanisms underlying lifestyle changes, health literacy, mHealth tools, and community-based multidisciplinary team interventions.

1. It is recommended that the authors clearly distinguish, at the beginning of the Methods and Results sections, between the following groups: the total RCT sample, the intervention group sample, those who actually participated in MMD, completers, app users, questionnaire respondents, and the interview sample. It is recommended to include a flowchart.

2. The researchers did not observe the implementation of the program nor interview healthcare professionals. The authors acknowledge in the Limitations section that this may have omitted important mechanisms influencing implementation.

3. The Introduction mentions that the MMD RCT reduced stroke risk by 61% at 12 months, and the Conclusion states that the combination of the RCT and this study demonstrates that

MMD supports lifestyle changes and reduces stroke risk in primary care. However, this paper is a process evaluation, not an outcome evaluation.

4. The authors acknowledge that they did not interview the control group, even though the control group in the RCT showed significant within-group improvements at 12 months. The authors should discuss the effects of non-specific interventions more systematically.

5. Table 1 also reveals a highly selective sample: 80% had more than 12 years of education, 99% were born in Europe, and 94% were still employed. How do the authors explain this?

6. The authors report that healthcare professionals completed all six sessions, indicating a “delivered dose” of 100%; however, the “received dose” was clearly insufficient: the average attendance rate per session was only 52.8%. The authors should explain whether the low attendance rate weakened or selectively influenced the intervention’s effects.

**Reviewer**	**2**
**Name**	**Renna, Nicolas**
**Affiliation**	**Hospital Español de Mendoza , Department of Cardiology**
**Date**	**21-Jul-2026**
**COI**	**None**

Thank you for the opportunity to review this interesting manuscript evaluating the implementation process of the "Make My Day" stroke prevention intervention. This is a well-conducted mixed-methods process evaluation performed alongside a randomized controlled trial and addresses an important aspect of complex lifestyle interventions that is often overlooked. The use of the MRC Process Evaluation Framework, the combination of quantitative and qualitative data, and the focus on implementation mechanisms represent important strengths of the study.

Overall, I found the manuscript well written and methodologically sound. However, several aspects require clarification and a more balanced interpretation before the manuscript can be considered for publication. My main concerns relate to the interpretation of implementation fidelity, the description of the mixed-methods integration, the handling of investigator reflexivity, and the generalizability of the findings.

Major comments

1. Please strengthen the rationale for this process evaluation beyond the previously published RCT.

The introduction explains why process evaluations are important for complex interventions, but it does not clearly explain which specific knowledge gaps remained after the effectiveness trial. The manuscript would benefit from explicitly stating what questions

regarding implementation, mechanisms of change, or contextual influences could not be answered by the RCT alone.

2. The integration of quantitative and qualitative findings requires further methodological detail.

The manuscript states that both datasets were analysed separately and subsequently integrated using a convergent mixed-methods approach. However, the integration process itself is only briefly described. Please explain:

1- how both datasets informed each other;

2- how discrepant findings were handled;

3- whether joint displays or integration matrices were used;

4- how the final synthesis was developed.

This information is important for readers interested in mixed-methods methodology.

3. Please provide additional information regarding reflexivity and investigator bias.

Members of the research team participated in data collection, interviews, field notes, and qualitative analysis. While this is common in qualitative research, the manuscript should describe in greater detail the procedures used to minimize potential investigator bias (e.g., reflexive discussions, independent coding, triangulation, audit procedures, or consensus meetings).

4. Interpretation of implementation fidelity should be more balanced.

The abstract and discussion state that overall adherence to the intervention was good. However, the reported findings indicate:

1- mean attendance of only 3.6 out of six sessions;

2- important variation in fidelity across sites;

3- adaptations of planned activities;

4- declining app use;

5- incomplete implementation of some intervention components.

These findings suggest a more nuanced interpretation of intervention fidelity. The wording throughout the manuscript should better reflect this variability.

5. The implications of the relatively low intervention exposure deserve further discussion.

Although all planned sessions were delivered, participant attendance was modest and only a subset completed the intervention. It would be valuable to discuss:

whether a minimum exposure ("dose") was considered necessary for behaviour change;

whether participants with greater attendance experienced different outcomes;

how incomplete exposure may influence interpretation of the identified mechanisms of change.

6. The manuscript should be more cautious when referring to "mechanisms of change."

Many conclusions regarding mechanisms are based on participants' perceptions and experiences rather than direct evidence demonstrating causal mechanisms. Consider using more cautious terminology such as "perceived mechanisms," "potential mechanisms," or "participants identified..." where appropriate.

7. Generalizability requires further discussion.

The study population consisted predominantly of participants with relatively high educational attainment, almost exclusively European-born individuals, and a high employment rate. In addition, recruitment of socioeconomically disadvantaged populations proved challenging.

These characteristics should be discussed more explicitly as they may limit transferability of the findings to more vulnerable populations where stroke prevention interventions are often most needed.

8. Please expand the practical implications for future implementation.

One of the major values of process evaluations is informing future implementation. The discussion would benefit from more concrete recommendations regarding how the intervention could be optimized based on the present findings (e.g., group composition, session structure, healthcare professional training, digital tools, or participant engagement strategies).

Minor comments

Consider explicitly mentioning in the abstract that the study followed the MRC Process Evaluation Framework.

The description of the Swedish healthcare reform could be shortened to allow more emphasis on the conceptual basis of the intervention itself.

Please clarify how the qualitative sample size (n = 20) was determined and whether thematic saturation was achieved.

More information regarding the healthcare professional training programme (duration, competency assessment, standardization) would improve reproducibility.

Recruitment through social media and newspaper advertisements may have introduced self-selection bias. This should be acknowledged more explicitly among the study limitations.

The role of the mobile application deserves further quantification. Given the relatively low usability score and declining use over time, it would be informative to discuss whether app engagement was associated with adherence or perceived behaviour change.

Consider summarizing the study strengths and limitations in a more concise and balanced manner within the discussion.

Overall assessment

This is a valuable study addressing an important implementation question in stroke prevention. The study is methodologically sound and has several notable strengths, including its mixed-methods design, prospective process evaluation, and use of an established implementation framework. The requested revisions mainly concern clarification of methodology, more balanced interpretation of the findings, and stronger discussion of implementation and generalizability.

I believe these revisions would substantially strengthen the manuscript.

VERSION 1 - AUTHOR RESPONSE

Response to edotor and reviewers:
Thank you for your time in providing thoughtful and valuable reflections and comments that have significantly improved the manuscript. We have reviewed all your comments and suggestions and considered them in our rewriting. We have rephrased and rewritten to further clarify and nuance the process and results throughout the paper including the abstract, method, and discussion.

Reviwer 1 comments	Response
1. It is recommended that the authors clearly distinguish, at the beginning of the Methods and Results sections, between the following groups: the total RCT sample, the intervention group sample, those who actually participated in MMD, completers, app users, questionnaire respondents, and the interview sample. It is recommended to include a flowchart.	Thank you for this comment.<br>We have added characteristics of both intervention group and the interview sample to the RCT sample in Table 1 on page 3.<br>Information on app use, questionnaire respondents, and interview sample is stated in Table 2 on page 7. Although we can see benefits with a flowchart all information is already in the tables and would therefore be repeating information. Replacing the Tables with a flowchart would leave out important data.
2. The researchers did not observe the implementation of the program nor interview healthcare professionals. The authors acknowledge in the Limitations section that this may have omitted important mechanisms influencing implementation.	Thank you for this comment. Interviews were performed with all healthcare professionals. However, due to the scope and aims of this study the interviews will be presented in a seprate study. The future study where the healthcare professionals perspectives are included may provide a complementary illustration of the mechanisms presented and discussed in the present study. A clarifying sentence has been added to the study design on page 3.
3. The Introduction mentions that the MMD RCT reduced stroke risk by 61% at	Thank you for highlighting this. The sentence has been rewritten to point to the

12 months, and the Conclusion states that the combination of the RCT and this study demonstrates that MMD supports lifestyle changes and reduces stroke risk in primary care. However, this paper is a process evaluation, not an outcome evaluation	two studies different contributions evaluating the process and the MMD intervention. This can be found in the conclusion on page 16.
4. The authors acknowledge that they did not interview the control group, even though the control group in the RCT showed significant within-group improvements at 12 months. The authors should discuss the effects of non-specific interventions more systematically.	Thank you for this important point. As these results were presented in the RCT they been problematised and discussed in-depth in the RCT-study (Johnsson et al, 2025). Complementing text has been added to the discussion citing this discussion on page 16.
5. Table 1 also reveals a highly selective sample: 80% had more than 12 years of education, 99% were born in Europe, and 94% were still employed. How do the authors explain this?	Despite using several recruitment strategies to reach a heterogeneous population, the participants recruited to the study were predominantly highly educated, born in Europe, and employed. The reasons for this are not fully known; however, it is possible that factors such as availability, work flexibility, familiarity with research participation, and other social and structural resources influenced willingness or ability to participate. Consequently, certain groups may be underrepresented in the sample. This issue and its implications for the interpretation and transferability of the findings reflected on on page 16 and are discussed in greater detail in the RCT study (Johnsson et al, 2025).
6. The authors report that healthcare professionals completed all six sessions, indicating a "delivered dose" of 100%; however, the "received dose" was clearly insufficient: the average attendance rate per session was only 52.8%. The authors should explain whether the low attendance rate weakened or selectively influenced the intervention's effects.	Thank you for this important point. A complementing paragraph has been added to the methodological considerations discussing this issue on page 16, and citing the discussion in the RCT-study where this also has been problematised and discussed.

Reviewer 2 comments	Response
1. Please strengthen the rationale for this process evaluation beyond the previously published RCT.<br><br>The introduction explains why process evaluations are important for complex interventions, but it does not clearly explain which specific knowledge gaps remained	Thank you for this important observation. While the RCT demonstrated the effectiveness of the *Make My Day* intervention in reducing stroke risk, it could not explain how and why the intervention generated change, how it was experienced by participants and providers, or how contextual factors influenced

after the effectiveness trial. The manuscript would benefit from explicitly stating what questions regarding implementation, mechanisms of change, or contextual influences could not be answered by the RCT alone.	implementation and outcomes. As *Make My Day* is a novel intervention, these aspects had not previously been investigated. We have therefore revised the Introduction to clarify the specific knowledge gaps addressed by this process evaluation and its added value beyond the effectiveness findings reported in the RCT study. This can be found on page 2
2. The integration of quantitative and qualitative findings requires further methodological detail.<br><br>The manuscript states that both datasets were analysed separately and subsequently integrated using a convergent mixed-methods approach. However, the integration process itself is only briefly described. Please explain:<br>1- how both datasets informed each other;<br>2- how discrepant findings were handled;<br>3- whether joint displays or integration matrices were used;<br>4- how the final synthesis was developed.<br><br>This information is important for readers interested in mixed-methods methodology.	Thank you for highlighting the need for greater methodological transparency regarding the integration process.<br>We have expanded the Synthesis section to provide a more detailed description of how quantitative and qualitative findings were integrated within the convergent mixed-methods design. Specifically, we clarify that the datasets were analysed independently and subsequently brought together utelising an integration matrix to facilitate systematic comparison across datasets. We describe how quantitative findings informed the interpretation of qualitative themes and vice versa, how areas of convergence, complementarity, and divergence were identified and how discrepant findings were examined and discussed within the research team rather than excluded. We have also clarified that the final synthesis was developed through an iterative process of comparing findings across datasets and discussing interpretations among the authors until consensus was reached.. These revisions provide greater transparency regarding the integration procedures and the development of the mixed-methods findings.<br>This can be found under synthesis on page 6
3. Please provide additional information regarding reflexivity and investigator bias. Members of the research team participated in data collection, interviews, field notes, and qualitative analysis. While this is common in qualitative research, the manuscript should describe in greater detail the procedures used to minimize potential investigator bias (e.g., reflexive discussions,	Thank you for raising this important point. A reflexivity paragraph has been added to the Methods section following the description of the data analysis to increase transparency regarding the researchers' roles and potential influence on the study. We describe the involvement of the research team in both data collection and analysis, including that only the first author participated in both phases. We also clarify

independent coding, triangulation, audit procedures, or consensus meetings).	the strategies used to minimise potential investigator bias, including reflexive discussions within the research team, triangulation of data sources, and consensus meetings during both data collection and analysis. This can be found on page 8
4. Interpretation of implementation fidelity should be more balanced.<br>The abstract and discussion state that overall adherence to the intervention was good. However, the reported findings indicate:<br>1- mean attendance of only 3.6 out of six sessions;<br>2- important variation in fidelity across sites;<br>3- adaptations of planned activities;<br>4- declining app use;<br>5- incomplete implementation of some intervention components	Adjustments have been made throughout the Abstract and Discussion to provide a more balanced interpretation of implementation fidelity. The Abstract has been revised to distinguish between overall adherence to the intervention manual and the observed variation in fidelity across sites, attendance rates, implementation of specific intervention components, and app engagement, page 1.<br>The Discussion now opens with a description of the variation in implementation fidelity across sites and the potential implications of these findings. Additional text has also been added regarding participant attendance, adaptations to intervention activities, declining app use over time, and incomplete implementation of some intervention components.On pages, 12, 15, and 16.
5. The implications of the relatively low intervention exposure deserve further discussion.<br>Although all planned sessions were delivered, participant attendance was modest and only a subset completed the intervention. It would be valuable to discuss:<br>whether a minimum exposure ("dose") was considered necessary for behaviour change;<br>whether participants with greater attendance experienced different outcomes;<br>how incomplete exposure may influence interpretation of the identified mechanisms of change	Thank you for this important point. A complementing paragraph has been added to the methodological section, which also refers to the discussion in the RCT where this issue is further addressed. Found on page 16<br><br>We agree that it would have been valuable to examine whether intervention attendance was associated with differences in stroke risk outcomes. However, attendance data were only available at the group level and not at the individual participant level, making such analyses impossible. This limitation and its implications for interpreting intervention exposure and mechanisms of change are now discussed and has been added to the new paragraph in the methodological considerations.
6. The manuscript should be more cautious when referring to "mechanisms of change." Many conclusions regarding mechanisms are based on participants' perceptions and	Adjustments have been made when discussing mechanisms of change as being perceived or identified by participants throughout the manuscripts.

experiences rather than direct evidence demonstrating causal mechanisms. Consider using more cautious terminology such as "perceived mechanisms," "potential mechanisms," or "participants identified..." where appropriate	
7. Generalizability requires further discussion.<br>The study population consisted predominantly of participants with relatively high educational attainment, almost exclusively European-born individuals, and a high employment rate. In addition, recruitment of socioeconomically disadvantaged populations proved challenging.<br><br>These characteristics should be discussed more explicitly as they may limit transferability of the findings to more vulnerable populations where stroke prevention interventions are often most needed	Thank you for this important comment. We have expanded the methodological considerations, found on page 16 to discuss the limited representation of participants with lower socioeconomic status, lower educational attainment, and non-European backgrounds, and how this may affect transferability of the findings. These recruitment challenges are recognised as an important limitation. In response, we are currently conducting a new study that includes adapted recruitment strategies and an intervention tailored to socioeconomically disadvantaged areas.
8. Please expand the practical implications for future implementation.<br><br>One of the major values of process evaluations is informing future implementation. The discussion would benefit from more concrete recommendations regarding how the intervention could be optimized based on the present findings (e.g., group composition, session structure, healthcare professional training, digital tools, or participant engagement strategies).	Thank you for this comment.<br>The conclusion with clinical implications has been rewritten with a clearer focus also entailing our concrete adjustments and new studies of MMD to improve and optimise future implementation.Found on pages 16-17
Minor comments<br>Consider explicitly mentioning in the abstract that the study followed the MRC Process Evaluation Framework.<br>The description of the Swedish healthcare reform could be shortened to allow more emphasis on the conceptual basis of the intervention itself.<br>Please clarify how the qualitative sample size (n = 20) was determined and whether thematic saturation was achieved.	Thank you for these comments.<br>The MRC has been added to the abstract.<br><br>We have shortened the text on Swedish healthcare reform and added text on the knowledge gap in the RCT prompting this process evaluation but chosen to keep the conceptual text on the MMD intervention in the method section to keep the alignment of the text. Found on page 2<br><br>Information has been added on sample size and selection which can be found under data collection, pages 5-6

More information regarding the healthcare professional training programme (duration, competency assessment, standardization) would improve reproducibility.<br>Recruitment through social media and newspaper advertisements may have introduced self-selection bias. This should be acknowledged more explicitly among the study limitations.	Thank you for this comment. Additional text has been added on the education regarding its duration over two weeks with both face-to-face and digital group sessions utilising a collaborative learning model. For more reading the citation to the study on the education, Jakobsson et al 2025, is listed, found on pages 3-4.
The role of the mobile application deserves further quantification. Given the relatively low usability score and declining use over time, it would be informative to discuss whether app engagement was associated with adherence or perceived behaviour change	Thank you for this comment. There is a separate study planned on the mobile application, quantifying its use, limitations and how it was perceived by the participants.
Consider summarizing the study strengths and limitations in a more concise and balanced manner within the discussion.	Thank you for this comment. The strengths and limitations section has been revised to provide a more concise and balanced summary of the study's methodological strengths and limitations and their implications for interpretation of the findings. Found on page 1 and 14-17.

VERSION 2 - REVIEW

**Reviewer**	**1**
**Name**	**Zhu, Luwen**
**Affiliation**	**Heilongjiang University of Chinese Medicine, Fourth Affiliated Hospital**
**Date**	**29-Aug-2026**
**COI**	

This manuscript can be published.

**Reviewer**	**2**
**Name**	**Renna, Nicolas**
**Affiliation**	**Hospital Español de Mendoza , Department of Cardiology**
**Date**	**04-Sep-2026**
**COI**	

The authors have satisfactorily addressed the major concerns raised in my previous review. The revised manuscript is substantially clearer and more balanced, particularly regarding intervention fidelity, participant exposure, reflexivity, transferability of the findings, and the distinction between observed findings and perceived mechanisms of change.

I have one remaining minor comment. In the methodological considerations, the authors state that the significant reduction in stroke risk despite relatively low attendance may suggest that, for some participants, “minimal exposure to the intervention might have been sufficient to initiate a change process.” This interpretation appears somewhat speculative. As attendance data were not available at the individual level and an exposure-response analysis could therefore not be performed, the present data cannot establish whether minimal intervention exposure was sufficient to produce behavioural or clinical change.

I suggest removing this statement or reframing it explicitly as a hypothesis that should be evaluated in future studies.

Apart from this minor point, I consider my previous concerns adequately addressed and believe the manuscript is suitable for publication after minor revision.

VERSION 2 - AUTHOR RESPONSE

Reviewer 2	Response to reviewer comment
In the methodological considerations, the authors state that the significant reduction in stroke risk despite relatively low attendance may suggest that, for some participants, “minimal exposure to the intervention might have been sufficient to initiate a change process.” This interpretation appears somewhat speculative. As attendance data were not available at the individual level and an exposure-response analysis could therefore not be performed, the present data cannot establish whether minimal intervention exposure was sufficient to produce behavioural or clinical change.<br>I suggest removing this statement or reframing it explicitly as a hypothesis that should be evaluated in future studies.	Thank you for this comment. We have revised accordingly adding text clarifying this is an assumption that needs further attention. ” Nonetheless, the IG exhibited a significant reduction in stroke risk, suggesting that, for some participants, minimal exposure to the intervention might have been sufficient to initiate a change process. However, as attendance data were not available at the individual level, an exposure-response analysis could not be performed, and the assumption needs further investigation.” This can be found on page 16.
