# Supplementary material for: Performing lifestyle changes and reducing stroke risk: a process evaluation of a stroke prevention intervention
Source: BMJ Open. 2026 Sep 30;16(9):e123012. doi: 10.1136/bmjopen-2026-123012 (PMC13629870; doi:10.1136/bmjopen-2026-123012)
Supplement: Supplementary file 2 [file bmjopen-16-9-s001.docx]

**Examples of questions for people at risk of stroke who have participated in the programme:**

(The questions under the bullet points are intended as possible follow-up questions depending on how informative/exhaustive the initial answer is)

- Can you tell us about your experiences of participating in the MMD program?
- Can you describe how it was in the beginning
- Was there a turnaround somewhere?
- Did you feel the same way at the end of the program?
- What did you think was the most important thing for you about the program – can you name a few things
- What was it like to absorb the information in the program?
- What can be improved?
- What was good?
- Did you experience any challenges during the program – can you tell us/describe
- What do you see as opportunities for improvement with the program?
- What were the weaknesses in the program's structure?
- What were the strengths of the setup?
- Can you reason about which themes and parts of the program contributed to supporting your work with the goals and change in your
- Can you give examples of how they contributed
- What does it look like today?
- Can you discuss which themes and parts of the programme did not help to support your work with the goals and change in your area?
- What themes and elements would have been needed to achieve change in your company?
- Can you tell us how the group contributed to or affected the program and any changes that you made,
- Can you describe some positive situations/experiences
- Can you tell us about any challenging situations/experiences
- Can you describe your lifestyle and activity patterns in relation to the program?
- Has there been any change? – can you tell me
- Can you tell us how you went about it?
- Can you describe if you feel any effects of the change
- Can you tell us how you reason about engaging activities
- - What do engaging activities entail for you?
- Can you describe or tell us about your engaging activities and in relation to the program?
- If there has been any change - can you tell me
- Can you describe your knowledge about stroke and health/illness in relation to the programme
- Did the programme contribute to your knowledge and if so, how?
- Have you and your family members discussed risk factors for stroke
- In what way have you reasoned?
- If no, how have you reasoned?
- Have you and your family members talked about your participation in MMD?
- If so, in what way?
- If not, was there any reason why?
- How did you experience the app as support in the change process?
- How can the app be improved?
- Can you tell us about the time after the programme and until now when you have worked independently
- What has worked well?
- Have you taken any tools from the program that you have used
- What challenges and difficulties have you experienced?
- How have you dealt with these
- Is there anything that you would like to add that we have not talked about or that you would like to clarify?
